# Contribution of NOTCH1 genetic variants to bicuspid aortic valve and other congenital lesions

**DOI:** 10.1136/heartjnl-2021-320428

**Published:** 2022-03-14

**Authors:** Radoslaw Marek Debiec, Stephen E Hamby, Peter D Jones, Kassem Safwan, Michael Sosin, Simon Lee Hetherington, David Sprigings, David Sharman, Kelvin Lee, Pegah Salahshouri, Nigel Wheeldon, Andrew Chukwuemeka, Vasiliki Boutziouka, Mohamed Elamin, Sue Coolman, Manish Asiani, Shireen Kharodia, Gregory J Skinner, Nilesh J Samani, Tom R Webb, Aidan P Bolger

**Affiliations:** 1 Department of Cardiovascular Sciences and NIHR Leicester Biomedical Research Centre, University of Leicester, College of Medicine Biological Sciences and Psychology, Leicester, UK; 2 East Midlands Congenital Heart Centre, Glenfield Hospital, University Hospitals of Leicester NHS Trust, Leicester, UK; 3 Department of Cardiology, University Hospitals of Leicester NHS Trust, Leicester, UK; 4 Department of Cardiology, Nottingham University Hospitals NHS Trust, Nottingham, UK; 5 Department of Cardiology, Kettering General Hospital NHS Foundation Trust, Kettering, UK; 6 Department fo Cardiology, Northampton General Hospital NHS Trust, Northampton, UK; 7 Lincolnshire Heart Centre, United Lincolnshire Hospitals NHS Trust, Lincoln, UK; 8 Department of Cardiology, West Suffolk NHS Foundation Trust, Bury Saint Edmunds, UK; 9 Cardiothoracic Centre, Northern General Hospital, Sheffield Teaching Hospitals NHS Trust, Sheffield, UK; 10 Departments of Cardiac Surgery and Cardiology, Hammersmith Hospital, Imperial College Healthcare NHS Trust, London, UK; 11 The Heart Centre, Royal Derby Hospital, University Hospitals of Derby and Burton NHS Foundation Trust, Derby, UK

**Keywords:** bicuspid aortic valve, genetics

## Abstract

**Introduction:**

Bicuspid aortic valve (BAV) affects 1% of the general population. *NOTCH1* was the first gene associated with BAV. The proportion of familial and sporadic BAV disease attributed to *NOTCH1* mutations has not been estimated.

**Aim:**

The aim of our study was to provide an estimate of familial and sporadic BAV disease attributable to *NOTCH1* mutations.

**Methods:**

The population of our study consisted of participants of the University of Leicester Bicuspid aoRtic vAlVe gEnetic research—8 pedigrees with multiple affected family members and 381 sporadic patients. All subjects underwent *NOTCH1* sequencing. A systematic literature search was performed in the NCBI PubMed database to identify publications reporting *NOTCH1* sequencing in context of congenital heart disease.

**Results:**

*NOTCH1* sequencing in 36 subjects from 8 pedigrees identified one variant c.873C>G/p.Tyr291* meeting the American College of Medical Genetics and Genomics criteria for pathogenicity. No pathogenic or likely pathogenic *NOTCH1* variants were identified in 381 sporadic patients. Literature review identified 64 relevant publication reporting *NOTCH1* sequencing in 528 pedigrees and 9449 sporadic subjects. After excluding families with syndromic disease pathogenic and likely pathogenic *NOTCH1* variants were detected in 9/435 (2.1%; 95% CI: 0.7% to 3.4%) of pedigrees and between 0.05% (95% CI: 0.005% to 0.10%) and 0.08% (95% CI: 0.02% to 0.13%) of sporadic patients. Incomplete penetrance of definitely pathogenic *NOTCH1* mutations was observed in almost half of reported pedigrees.

**Conclusions:**

Pathogenic and likely pathogenic *NOTCH1* genetic variants explain 2% of familial and <0.1% of sporadic BAV disease and are more likely to associate with tetralogy of Fallot and hypoplastic left heart.

## Introduction

Bicuspid aortic valve (BAV) is the most common valvular congenital heart defect, affecting 1% of the general population. BAV commonly associates with coarctation of aorta (CoA) and ventricular septal defect (VSD).^1^ BAV can lead to clinical complications including aortic valve disease, thoracic aortic aneurysm (TAA) and infective endocarditis.^2^ BAV clusters within families following an autosomal dominant pattern of inheritance and its heritability has been estimated between 47% and 89%.^3 4^

*NOTCH1* (MIM# 190198) was the first gene associated with inherited aortic valve disease.^5^ Garg *et al* described two pedigrees, where predominantly left-sided, cardiac lesions (including BAV) co-segregated with damaging *NOTCH1* mutations.^5^ Subsequently, *NOTCH1* mutations were associated with other left (eg, CoA, hypoplastic left heart syndrome (HLHS)) and right-sided (eg, pulmonary stenosis, pulmonary atresia) congenital defects as well as tetralogy of Fallot (ToF) and Adams-Oliver syndrome (AOS).^6–8^

The report of Garg *et al* nurtured further sequencing studies. Mohamed *et al* reported two likely pathogenic mutations in a sample of 48 patients with BAV.^9^ McKeller *et al* reported missense *NOTCH1* variants in 10.4% of patients with concomitant BAV and TAA, whereas McBride *et al* found potentially damaging *NOTCH1* mutations in 6.6% of subjects with left ventricular outflow tract disease.^10 11^ The uncertain functional status of identified genetic variants informed further sequencing studies in patients with ‘more severe’ phenotypes including CoA, and BAV associated with aortic root phenotype.^12 13^ Finally, variable analytical approaches were applied including analysis of *NOTCH1* variants under recessive model of inheritance,^14^ sex-specific analysis^15^ or burden testing.^16^ The use of various phenotype definitions, research approaches and analytical methods made interpretation of these result difficult and the proportion of BAV disease attributed to *NOTCH1* mutations has not been estimated. This lack of knowledge may hinder use of *NOTCH1* sequencing for the purpose of genetic counselling.

To provide an accurate estimate of familial and sporadic BAV disease attributed to pathogenic *NOTCH1* mutations, we performed sequencing of *NOTCH1* in a cohort of familial and sporadic cases of BAV as well as a systematic review of the pathogenicity of previously reported variants.

## Aim and hypothesis

We hypothesised that the burden of BAV disease due to *NOTCH1* mutations had been overestimated. The main aim of our study was to provide an estimate of familial and sporadic BAV disease attributable to *NOTCH1* mutations. The secondary aim was to identify a pattern of association of *NOTCH1* mutations with congenital cardiac lesions. The tertiary aim was a critical review of the available literature and an overview of the contribution of *NOTCH1* variants in congenital heart disease.

## Methods

### Study participants

The population of our study were participants of the University of Leicester Bicuspid aoRtic vAlVe gEnetic research (BRAVE)—an ongoing, multicentre recruitment of patients with BAV disease. Participants have been identified by review of clinic and discharge letters. Diagnosis of BAV was confirmed by review of cardiac MRI (cMRI), trans-oesophageal echocardiography (TOE) or unequivocal transthoracic echocardiography. Where possible, intra-operative description of valve morphology was obtained. Demographic and clinical data were collected using purposefully designed questionnaires (REDCap). Individuals with positive familial history were offered cascade echocardiographic screening.

### Laboratory processing of blood

A venous blood sample (30 mL) was obtained from each participant for the purpose of DNA extraction (QIAsymphony DSP DNA Midi kit/automatic device). All samples were assessed for purity by checking 260/280 nm and 260/230 nm absorbance ratios and diluted to a concentration of 100 ng/µL.

### Exome sequencing in pedigrees

DNA samples from subjects with familial BAV underwent whole exome sequencing (WES) (BGI, China). Library capture was performed using BGI Exome (59M) capture kit. Exome sequencing was performed using the Illumina Hiseq platform. Sequence reads for each sample were aligned to the reference genome (hg19) using Burrows-Wheeler Aligner V0.7.15. Variant calling was carried out using the HaplotypeCaller of GATK (V.3.6) (Broad Institute).

### NOTCH1 sequencing in sporadic subjects

DNA samples from patients with sporadic BAV underwent targeted *NOTCH1* sequencing (Source BioScience, UK). Libraries were prepared using the KAPA Hyperplus kit (Roche), hybridised using the SeqCap Hybridisation kit (Roche) and sequenced with the Illumina MiSeq.

### Variant analysis

Variant calls were filtered according to quality control metrics: quality by depth, Fisher strand, RMSMapping quality and Read position rank sum (testing for distance from end of read). Annotation was carried out using Ensembl Variant Effect Pedictor^17^ and included assignment of amino acid changes, gnomAD allele frequency and the Combined Annotation Dependent Depletion (CADD) functional prediction score.^18^

### NOTCH1 variant burden testing

A *NOTCH1* variant burden testing was performed following the protocol described by Gillis *et al* 16 (see online supplemental materials). The allele counts from sequencing of sporadic patients recruited to BRAVE study were compared with the counts obtained from the control population of gnomAD (gnomAD database V.2.1.1).^19^ Both datasets were filtered to include variants with a minor allele frequency (MAF) of <0.0001 and/or MAF frequency of <0.001 and a CADD score of >20.0. Allele counts in the control dataset were scaled to the mean total allele count. Fisher’s exact test was used to assess for enrichment of variants in the cases over the controls.

10.1136/heartjnl-2021-320428.supp1 Supplementary data

### Literature search

A systematic literature search was performed in the NCBI PubMed database using “*NOTCH1* aortic valve”, “*NOTCH1* mutation” and “*NOTCH1* variant” terms. The search included all manuscripts published before 31 October 2020 reporting *NOTCH1* sequencing in context of congenital heart disease where BAV can be a phenotypic feature. No age or ethnicity exclusion criteria were applied as BAV is an inherited condition and affects all ethnicities. Only original papers written in English and containing information on sequencing of *NOTCH1* variants were considered. The compiled bibliography of all papers identified as suitable was further reviewed to identify other qualifying papers.

All reported *NOTCH1* variants were recorded and annotated for gnomAD MAF.^19^ Variants with MAF <0.001 were assessed for pathogenicity in accordance with the American College of Medical Genetics and Genomics (ACMG) criteria^20^ using VarSome,^21^ ClinVar^22^ and review of the source publications.

For the purpose of summary analysis, the subjects of each of the studies were divided into familial and sporadic cases. Familial cases were defined as affected probands, who had at least one objectively confirmed, affected first-degree or second-degree relative. Sporadic cases were defined as individual patients with no reported first-degree or second-degree affected relatives. Detailed information on criteria used for classification of familial and sporadic patients are provided in the online supplemental materials.

## Results

### Patient characteristics

**Figure 1:**
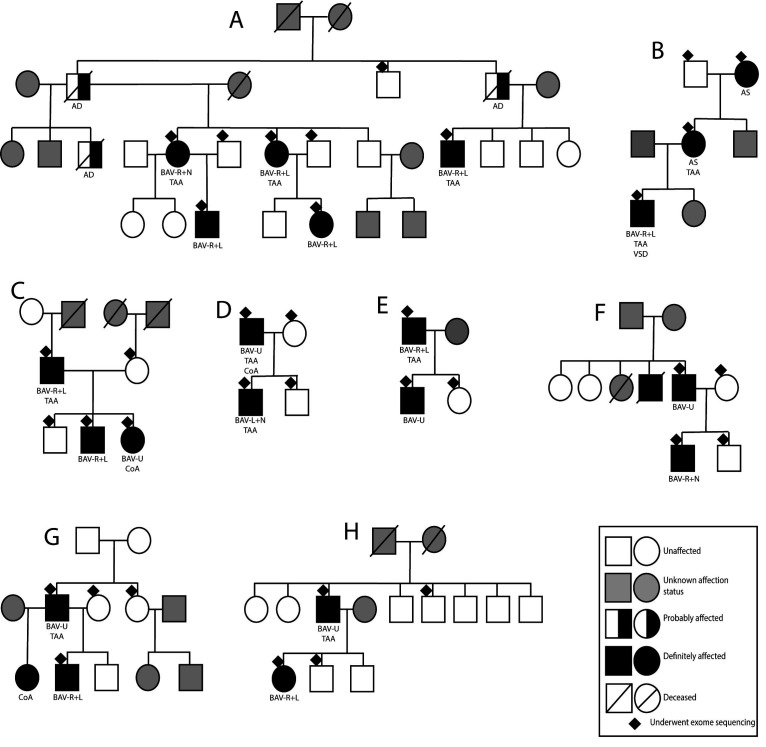
Family trees and phenotypic information of eight pedigrees with familial form of BAV disease. AD, aortic dissection; AS, aortic valve stenosis; BAV, bicuspid aortic valve (R+L, right-coronary and left-coronary cusp fusion; R+N, right-coronary and non-coronary leaflet fusion; U, unknown leaflet fusion pattern); CoA, coarctation of aorta; TAA, thoracic aortic aneurysm; VSD, ventricular septal defect.

The group of familial BAV disease comprised 8 families and included 36 individuals (21 affected and 15 unaffected). Clinical phenotypic information and family trees are presented in figure 1 and table 1.

**Table 1:** Clinical characteristics of patients

		Familial patients (n=19 BAV, n=2 AS*)	Sporadic patients (n=381)	P value
Males, n (%)		14 (67)	277 (73)	0.6165
Age at recruitment (median)		40.2	52.1	0.0216
White ethnicity		21 (100)	300 (79)	0.0111
Leaflet fusion pattern, n (%)	RL	10 (52)	231 (61)	1.0
	RN	2 (11)	68 (18)	0.7417
	LN	1 (5)	12 (3)	0.4188
	NA	6 (32)	70 (18)	–
TAA, n (%)		11 (52)	137 (36)	0.1629
CoA, n (%)		2 (10)	57 (15)	0.7521
VSD, n (%)		1 (5)	15 (4)	0.5833

P values calculated with the Fisher’s exact test for the comparison between categorical variables and with the Mann-Whitney U test for the group comparison of continuous variables.

*Two individuals diagnosed with aortic stenosis but carriers of pathogenic p.Tyr291*/c.873C>G *NOTCH1* variant.

AS, aortic stenosis; BAV, bicuspid aortic valve; CoA, coarctation of aorta; LN, fusion of left-coronary and non-coronary cusps; NA, leaflet fusion pattern not available; RL, fusion of right-coronary and left-coronary cusp; RN, fusion of right-coronary and non-coronary cusps; TAA, thoracic aortic aneurysm; VSD, ventricular septal defect.

Of the 21 affected subjects, 19 (90%) had an unequivocal diagnosis of BAV and 2 (9.5%) subjects (figure 1, pedigree B) had a diagnosis of aortic stenosis (AS) but were counted as affected due to the autosomal dominant transmission of a pathogenic *NOTCH1* mutation.^23^ In all pedigrees, the inheritance pattern was consistent with an autosomal dominant transmission. Among affected subjects, 14 of 21 individuals (67%) were male. Information about aortic valve cusp fusion pattern was available for 13 of 19 individuals (68%). Right-coronary and left-coronary cusp fusion pattern was observed in 10 (52%) individuals (table 1). TAA was diagnosed in 11 (52%) participants (8 males and 3 females). Two had CoA and one subjects had a VSD (figure 1).

The subgroup of individuals with sporadic BAV comprised 381 subjects. In 176 individuals (46%), the diagnosis of BAV was made using cMRI, in 55 (14%) by means of TOE, 105 (28%) patients were diagnosed by means of transthoracic echocardiogram and in 12 (3%) using a CT-aortogram; the remaining patients were diagnosed based on surgical description of the valve. Altogether, surgical description of the valve was available for 129 patients (34%). BAV with fusion of the right-coronary and left-coronary cusps was present in 231 (61%) patients, 68 subjects (18%) had a right-coronary and non-coronary cusp leaflet fusion and 12 had left-coronary and non-coronary leaflet fusion; in the remaining 70 subjects the pattern could not be determined (table 1). There was a predominance of males among the sporadic subjects (n=277, 73%) with male-to-female ratio of 2.7. The prevalence of associated phenotypes was: TAA (defined as aortic diameter ≥40 mm at any point of the aorta or previous TAA surgery) n=137 (35%), CoA n=57 (15%) and VSD n=15 (4%) (table 1).

### Exome sequencing of pedigrees

WES in 36 subjects from 8 pedigrees revealed 13 *NOTCH1* genetic variants (11 intronic variants, 1 synonymous variant and 1 nonsense variant) segregating with disease (table 2). Of these variants, only c.873C>G/p.Tyr291* in pedigree B (figure 1) met the ACMG criteria for pathogenicity. We previously reported this pedigree with more detailed phenotypic description.^23^

**Table 2:** Genetic variants co-segregating with affection status within the pedigrees with familial form of BAV disease

Variant ID	Pedigree ID	Location	MAF (gnomAD)	Function	Pathogenicity class
NA	B	p.Tyr291*/c.873C>G	–	Stop gained	Pathogenic
rs2229975	C	p.Pro284Pro/c.852G>A	0.1335	Synonymous variant	Benign
rs3812605	H	c.3171+220A>G	0.6629	Intron variant	Uncertain significance
rs11145764	E	c.4015-73G>A	0.4800	Intron variant	Benign
rs3124598	E	c.2970-31A>G	0.6431	intron variant	Benign
rs9411208	C	c.1441+7C>T	0.5782	Intron variant	Benign
rs11145765	D	c.3171+42G>A	0.09654	Intron variant	Benign
rs3124999	H	c.5639-174G>A	0.4420	Intron variant	Benign
rs3124603	B	c.1670-9A>G	0.4656	Intron variant	Benign
rs4880100	B	c.1556-133A>G	0.4746	Intron variant	Benign
rs10781498	B	c.1555+102C>T	0.4246	Intron variant	Benign
rs11145767	B	c.1555+10A>G	0.000004923	Intron variant	Benign
rs3125009	B	c.1100-140G>A	0.4267	Intron variant	Benign

BAV, bicuspid aortic valve; MAF, minor allele frequency; NA, not available.

### Sequencing of *NOTCH1* in sporadic cases

Sequencing of *NOTCH1* in individuals with sporadic BAV disease and filtering to a MAF frequency of <0.001 and CADD score >20 or MAF <0.0001 identified nine variants (table 3). None of the variants met ACMG criteria for classification as pathogenic. Rare variant burden testing compared with gnomAD controls^16^ showed no significant difference (9/762 alleles in cases compared with 4376/225 370 alleles in controls; p=0.15).

**Table 3:** NOTCH1 genetic variants (MAF frequency of <0.001 and CADD score >20 or MAF <0.0001) identified among 381 patients with sporadic BAV disease

Variant ID	Location	MAF (gnomAD)	Coding change	Protein change	CADD	Pathogenicity class
rs199652954	chr9:139 395 162	0.00007	c.5776C>T	p.Arg1926Cys	32	Likely benign
rs368400902	chr9:139 401 182	0.00003	c.3887G>A	p.Arg1296His	29.0	Likely benign
rs375119074	chr9:139 391 041	0.000004	c.7150C>G	p.Gln2384Glu	18.49	Uncertain significance
rs1166328821	chr9:139 399 309	–	c.4834G>A	p.Gly1612Ser	23.6	Uncertain significance
rs367825691	chr9:139 413 921	0.0001	c.839A>G	p.Asn280Ser	23.9	Likely benign
rs543533126	chr9:139 391 893	0.00003	c.6298A>G	p.Ile2100Val	21.8	Likely benign
rs375065108	chr9:139 417 517	0.00005	c.527G>A	p.Arg176Gln	21.2	Benign
rs1334842062	chr9:139 395 258	0.000004	c.5680G>A	p.Gly1894Ser	25.1	Uncertain significance
rs1212259128	chr9:139 418 258	0.000008	c.314C>G	p.Ala105Gly	18.97	Uncertain significance

BAV, bicuspid aortic valve; MAF, minor allele frequency.

### Literature review

**Figure 2:**
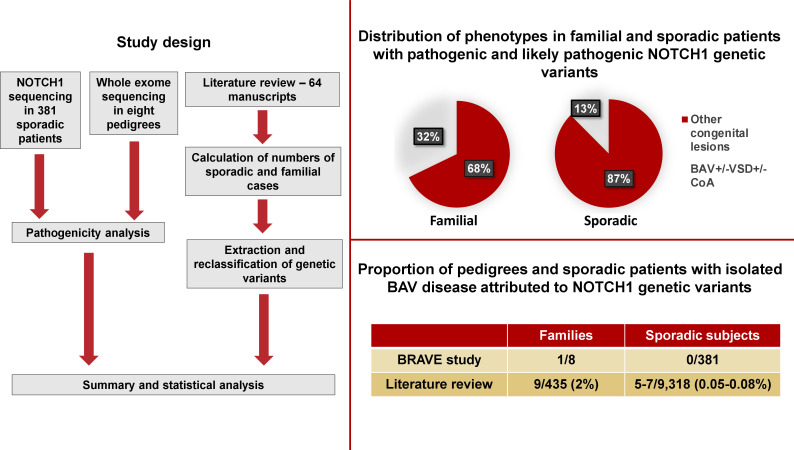
Study design and key findings. BAV, bicuspid aortic valve; BRAVE, Bicuspid aoRtic vAlVe gEnetic research; CoA, coarctation of aorta; VSD, ventricular septal defect.

A literature search in the PubMed database using the terms “*NOTCH1* aortic valve”, “*NOTCH1* mutation” and “*NOTCH1* variant” performed on 31 October 2020 returned 106, 1754 and 206 publications, respectively. There were 1858 unique references of which 1759 were rejected based on the title and the remaining assessed based on the abstract. Fifty-four manuscript met the inclusion criteria and 10 publications were identified through review of bibliography (figure 2 and online supplemental table 1).

Twenty-five studies focused on sequencing of sporadic subjects, 19 studies sequenced individuals from pedigrees with multiple affected individuals and 15 studies used a mixed design of sequencing both extended pedigrees as well as sporadic subjects. Altogether, the publications reported *NOTCH1* sequencing in subjects from 528 different pedigrees and 4669 sporadic cases. In addition, four studies focused on investigating de novo mutations by sequencing trios of affected proband and unaffected parents (4780 trios) (online supplemental table 1; no: 56, 58, 62, 63).

The included manuscripts reported pathogenic and likely pathogenic *NOTCH1* genetic variants in 89 individual patients carrying 74 unique genetic variants (online supplemental table 2 and figure 1). Of the recurring variants, only rs774966208 (c.578G>C, p.Gly193Ala) and rs1228192276 (c.428C>T, p.Pro143Leu) were reported in more than two individuals and both were assigned likely pathogenic ACMG status. Variant rs774966208 was reported in five patients with ToF and rs1228192276 in three patients with ToF (online supplemental table 1; no: 36). Of the 89 patients with pathogenic and likely pathogenic *NOTCH1* variants, 21 were reported in the context of syndromic disease including AOS (n=19), Shone complex (n=1) and 1 in a patient in whom congenital AS co-existed with myoclonic epilepsy and learning difficulties (online supplemental table 1; no: 9, 13, 32, 43, 47, 62). Of the remaining 68 non-syndromic individuals, 28 (42%) were reported in context of familial disease and 40 (58%) in context of sporadic disease.

Analysis of the phenotypic spectrum observed within the 28 affected pedigrees (harbouring pathogenic and likely pathogenic variants) revealed that in the majority *NOTCH1* mutations were associated with a wide range of congenital heart diseases including complex lesions such as ToF, truncus arteriosus or HLHS (online supplemental table 1; no: 1, 7, 17, 19, 24, 41, 50). In seven of these pedigrees, at least one individual was affected by ToF, in seven other by HLHS and two families had individuals with tricuspid valve atresia resulting in hypoplastic right ventricle. Phenotypic information were not available for three pedigrees. Only in nine pedigrees were the pathogenic and likely pathogenic *NOTCH1* variants associated with simple congenital phenotypes like isolated BAV, VSD or CoA (figure 2).

Of the 25 affected pedigrees (non-syndromic disease) harbouring definitely pathogenic mutations (stop mutations, frame shift, splice variants leading to loss of whole exomes), complete penetrance was observed in 11 pedigrees, incomplete penetrance was observed in 10 pedigrees (38 affected subject of 56 confirmed carriers of mutations in 10 pedigrees); detailed information/family trees were not available for 4 pedigrees.

Among the 40 sporadic patients harbouring pathogenic and likely pathogenic *NOTCH1* mutations, 25 had ToF and 8 had HLHS (online supplemental table 1; no: 5, 17, 25, 35, 36, 42, 62, 63). Detailed phenotypic information was not provided for two individuals. In only five sporadic participants were the *NOTCH1* pathogenic or likely pathogenic mutations reported in context of isolated congenital aortic valve or aortic disease: one patient had congenital AS, two patients BAV associated with TAA, one patient CoA and one patient had subvalvular AS with hypoplastic aorta and CoA (online supplemental table 1; no: 42, 43, 51, 63) (figure 2).

After excluding families with syndromic disease, pathogenic and likely pathogenic *NOTCH1* variants were detected in 28/435 (6.4%; 95% CI: 4.1% to 8.7%) of pedigrees with a wide spectrum of congenital cardiac lesions. Only in nine pedigrees, 9/435 (2.1%; 95% CI: 0.7% to 3.4%) *NOTCH1* pathogenic and likely pathogenic variants were associated with BAV and/or other simple lesions like isolated VSD or CoA.

Among non-syndromic sporadic cases pathogenic and likely pathogenic, *NOTCH1* mutations were responsible for 0.4% (95% CI: 0.3% to 0.6%) of the disease (40/9318). However, of the 40 patients 33 had either ToF or HLHS and a detailed phenotype was missing for two individuals. Therefore, the actual prevalence of *NOTCH1* pathogenic or likely pathogenic variants associated with sporadic BAV disease or BAV associated with simple lesions like isolated CoA or VSD was between 0.05% (95% CI: 0.005% to 0.10%; 5/9318) and 0.08% (95% CI: 0.02% to 0.13%; 7/9318), respectively.

## Discussion

In this study, we present results of *NOTCH1* sequencing of 8 pedigrees with familial BAV and 381 patients with sporadic BAV as well as results of a systematic review of *NOTCH1* sequencing in 528 pedigrees with familial congenital cardiac lesions and 9449 sporadic cases of congenital heart disease. The main finding from our study is that the pathogenic and likely pathogenic *NOTCH1* genetic variants explain only a small proportion of familial (2%) and sporadic (<0.10%) BAV disease. In non-syndromic familial and sporadic forms, *NOTCH1* mutations are more commonly associated with more complex congenital phenotypes and ToF and HLHS in particular.

WES of eight extended pedigrees recruited by our group identified a single *NOTCH1* pathogenic variant.^23^ There was a significant phenotypic heterogeneity within the carriers of the mutation with the phenotypes including BAV, AS of a trileaflet valve, TAA and VSD.^23^ We did not identify any pathogenic or likely pathogenic *NOTCH1* genetic variants in 381 participants with sporadic BAV. In the majority of BRAVE study participants, BAV was diagnosed using high-resolution imaging modalities (cMRI, TOE and intra-operative inspection of valve). We performed cascade echocardiographic screening in cases suspected of familial transmission. In this way, we have ensured accurate distinction between familial and sporadic cases. We have applied stringent and uniform criteria to categorise subjects reported in the literature.

Our findings are consistent with previous attempts of sequencing *NOTCH1* variants in large populations of individuals with BAV disease. Variant burden testing of NOTCH1 in 441 patients with BAV and TAA compared with ExAC controls showed enrichment in controls rather than cases.^16^ Burden testing performed in 60 patients with hereditary TAA associated with BAV compared with European controls found no difference in the proportion of qualifying *NOTCH1* variants.^24^

The second important finding form our study concerns the frequency of incomplete penetrance of *NOTCH1* mutations. This phenomenon was observed in almost half of the families carrying definitely pathogenic *NOTCH1* mutations and almost one-third of carriers of pathogenic or likely pathogenic variants had no clinical phenotype.

The results of our study indicate that sequencing of *NOTCH1* should be considered in familial and sporadic cases of AOS and sporadic and familial cases of ToF and HLHS.^7 25^ however, only around 6% of these will yield a positive result. *NOTCH1* sequencing may be considered in pedigrees with BAV and associated simple congenital phenotypes (CoA and VSD) with an expected yield of 2%. Caution has to be used when applying variant filtering in extended pedigrees to allow for incomplete penetrance. Finally, sequencing of *NOTCH1* in sporadic subjects with isolated BAV with or without TAA is not likely to identify pathogenic *NOTCH1* variants.

Our data have to be interpreted in context of possible methodological biases. Our analysis of *NOTCH1* mutation frequency is based on the number of variants that we annotated as pathogenic. However, a relatively large proportion of variants were classified as being of uncertain significance based on available evidence and contribution of these variants to disease cannot be excluded. Also, the segregation analysis of the four variants of unknown significance detected in the sporadic patients from BRAVE study was not possible as their relatives had not been recruited to the study.

The proportion of pedigrees with familial form of BAV disease in which *NOTCH1* nonsense, frameshift and splice variants were reported is likely to be overestimated due to non-reporting bias. It is possible that many more pedigrees with familial BAV had undergone sequencing for *NOTCH1* genetic variants but results of these efforts remain unpublished if no causative genetic variants were identified.

Sporadic patients, who reported no familial history of the disease, have not undergone cascade echocardiographic screening. This could result in non-detection of familial cases in our sporadic cohort.

Majority of the reported familial and sporadic patients, including the BRAVE study cohort, were of white ethnic origin.

In summary, the large sequencing effort in pedigrees and sporadic subjects with BAV combined with systematic literature review showed that *NOTCH1* mutations can be found in around 6% of families of white ethnic origin, with multiple congenital cardiac defects (including BAV) and around 2% of pedigrees with isolated familial BAV and/or CoA/VSD. Pathogenic *NOTCH1* variants explain <0.10% of isolated sporadic BAV cases of white ethnic origin. Further research in population of other ethnicities are necessary to confirm these estimates. Our study also indicates that further research is necessary to explain the incomplete penetrance of *NOTCH1* mutations.

**Key messages**

**What is already known on this subject?**

- Previous research suggested that pathogenic *NOTCH1* mutations explain up to 5% of all bicuspid aortic valve (BAV) disease.

**What might this study add?**

- Our data show that pathogenic *NOTCH1* mutations can be found in 2% of pedigrees with isolated familial BAV and only in <0.10% of sporadic BAV cases.

**How might this impact on clinical practice?**

- Our study may facilitate the use of *NOTCH1* sequencing in genetic counselling by providing the knowledge of the phenotypes associated with *NOTCH1* mutations as well as likelihood of detecting a mutation in patients undergoing genetic screening.

## Data Availability

Data are available on reasonable request. For data availability enquiries, please contact the corresponding author.
