# Supplementary material for: Contribution of NOTCH1 genetic variants to bicuspid aortic valve and other congenital lesions
Source: Heart. 2022 Mar 14;108(14):1114–20. doi: 10.1136/heartjnl-2021-320428 (PMC9240330; doi:10.1136/heartjnl-2021-320428)
Supplement: Supplementary data [file heartjnl-2021-320428supp001.pdf]

Supplementary materials

Contribution of NOTCH1 genetic variants to bicuspid aortic valve and other congenital lesions

CLASSIFICATION OF FAMILIAL AND SPORADIC CASES

Brave study

All subjects recruited to the BRAVE study underwent detailed phenotyping using purposefully designed, electronic questionnaire, which included information about cardiovascular health in first and second degree relatives. Individuals, who reported presence of BAV or any of the associated conditions (AS, CoA, VSD or any other form of congenital heart disease) or history of relevant cardiovascular procedures (aortic valve replacement, surgery for aortic aneurysm or congenital heart defect) in first or second degree relatives were offered a cascade echocardiographic screening. In brief: the proband was provided with invitation letters and study information leaflets with reply slips and asked to distribute these among first and second degree relatives. Upon returning the reply slips the relatives were invited for a study visit which included echocardiographic screening (if no previous, relevant cardiac imaging had been done). All attending relatives were recruited to the BRAVE study. Pedigrees where multiple affected members identified through interview or through cascade familial screening were considered as familial cases. Probands with negative familial history or, where the echocardiographic screening excluded presence of BAV or associated phenotypes in relatives, were considered sporadic.

Literature review

For the purpose of summary analysis of the literature review, the subjects of each of the included studies were divided into familial and sporadic cases. Individuals were considered to represent familial form of the disease if the affected proband had at least one objectively confirmed, clinically affected first or second degree relative. This definition included entire pedigrees with multiple affected and unaffected subjects as well as singulars, where further recruitment of other family members would allow for analysis of co-segregation of genotype and phenotype. In addition, cases with pathogenic or likely pathogenic mutations, where unaffected relatives were also carriers of the same variant, were also considered as familial. Sporadic cases were defined as individual patients with no reported first or second degree affected relatives. This definition also included patients, for whom cascade clinical screening or information about familial history was not available.

Debiec RM, *et al. Heart* 2022; 108:1114–1120. doi: 10.1136/heartjnl-2021-320428

NOTCH1 variant burden testing

A method previously described by Gillis et al. (1) was used for NOTCH1 variant burden testing. The counts of rare alleles meeting the criteria of MAF <0.0001 and/or MAF frequency of <0.001 and a CADD score of >20.0 was compared to counts obtained from the control population of GnomAD. GnomAD is a database containing information on frequency of genomic variants based on 125,748 exome sequences and 15,708 whole-genome sequences. The data come from aggregation of information on individual, non-related patients recruited as part of various disease-specific and population genetic studies. For further information please see https://gnomad.broadinstitute.org/.

1) Gillis E, Kumar AA, Luyckx I, Preuss C, Cannaerts E, van de Beek G, et al. Candidate Gene Resequencing in a Large Bicuspid Aortic Valve-Associated Thoracic Aortic Aneurysm Cohort: SMAD6 as an Important Contributor. Front Physiol. 2017;8:400.

Supplementary Table 1. Manuscripts included in the literature review

No	PMID	Title	Reference
1	16025100	Mutations in NOTCH1 cause aortic valve disease.	Nature. 2005;437:270-4.
2	16729972	Novel missense mutations (p.T596M and p.P1797H) in NOTCH1 in patients with bicuspid aortic valve.	Biochem Biophys Res Commun. 2006 ;345:1460-5.
3	17662764	Novel NOTCH1 mutations in patients with bicuspid aortic valve disease and thoracic aortic aneurysms.	J Thorac Cardiovasc Surg. 2007 ;134:290-6.
4	18593716	NOTCH1 mutations in individuals with left ventricular outflow tract malformations reduce ligand-induced signalling.	Hum Mol Genet. 2008 ;17:2886-93.
5	21457232	Identification of de novo mutations and rare variants in hypoplastic left heart syndrome.	Clin Genet. 2012 Jun;81(6):542-54.
6	23102684	Genotype-phenotype correlation in patients with bicuspid aortic valve and aneurysm.	J Thorac Cardiovasc Surg. 2013;146:158-165.e1.
7	23578328	Sequencing of NOTCH1, GATA5, TGFBR1 and TGFBR2 genes in familial cases of bicuspid aortic valve.	BMC Med Genet. 2013;14:44.
8	24418111	Variants in the NOTCH1 gene in patients with aortic coarctation.	Congenit Heart Dis. 2014;9:391-6.
9	25132448	Mutations in NOTCH1 cause Adams-Oliver syndrome.	Am J Hum Genet. 2014;95:275-84.
10	25260786	Use of a targeted, combinatorial next-generation sequencing approach for the study of bicuspid aortic valve.	BMC Med Genomics. 2014;7:56.
11	25776230	Ehlers-Danlos syndrome type IV is associated with a novel G984R COL3A1 mutation.	Mol Med Rep. 2015;12:1119-24.
12	25907466	Performant Mutation Identification Using Targeted Next-Generation Sequencing of 14 Thoracic Aortic Aneurysm Genes.	Hum Mutat. 2015;36:808-14.
13	25963545	Haploinsufficiency of the NOTCH1 Receptor as a Cause of Adams-Oliver Syndrome With Variable Cardiac Anomalies.	Circ Cardiovasc Genet. 2015;8:572-581.
14	26164125	Compound heterozygous NOTCH1 mutations underlie impaired cardiogenesis in a patient with hypoplastic left heart syndrome.	Hum Genet. 2015;134:1003-11.
15	26188975	Routine Genetic Testing for Thoracic Aortic Aneurysm and Dissection in a Clinical Setting.	Ann Thorac Surg. 2015;100:1604-11.
16	26708639	Identification of Gender-Specific Genetic Variants in Patients With Bicuspid Aortic Valve.	Am J Cardiol. 2016;117:420-6.
17	26820064	Cardiovascular malformations caused by NOTCH1 mutations do not keep left: data on 428 probands with left-sided CHD and their families.	Genet Med. 2016;18:914-23.
18	27058611	De Novo and Rare Variants at Multiple Loci Support the Oligogenic Origins of Atrioventricular Septal Heart Defects.	PLoS Genet. 2016;12:e1005963.
19	27760138	Family Based Whole Exome Sequencing Reveals the Multifaceted Role of Notch Signaling in Congenital Heart Disease.	PLoS Genet. 2016;12:e1006335.
20	27989580	The promises and challenges of exome sequencing in familial, non-syndromic congenital heart disease.	Int J Cardiol. 2017;230:155-163.
21	28246602	NOTCH1 Mutations in Aortic Stenosis: Association with Osteoprotegerin/RANK/RANKL.	Biomed Res Int. 2017;2017:6917907.
22	28387797	Genetic abnormalities in bicuspid aortic valve root phenotype: preliminary results.	Eur J Cardiothorac Surg. 2017;52:156-162.
23	28446798	Novel missense mutation in DLL4 in a Japanese sporadic case of Adams-Oliver syndrome.	J Hum Genet. 2017;62:851-855.
24	28473349	Use of Clinical Exome Sequencing in Isolated Congenital Heart Disease.	Circ Cardiovasc Genet. 2017;10:e001581.
25	28608148	Hypoplastic Left Heart Syndrome Sequencing Reveals a Novel NOTCH1 Mutation in a Family with Single Ventricle Defects.	Pediatr Cardiol. 2017;38:1232-1240.
26	28649221	NOTCH 1 Mutation in a Patient with Spontaneous and Recurrent Dissections of Extracranial Arteries.	Front Neurol. 2017;8:245.
27	28659821	Candidate Gene Resequencing in a Large Bicuspid Aortic Valve-	Front Physiol. 2017;8:400.

Debiec RM, *et al. Heart* 2022; 108:1114–1120. doi: 10.1136/heartjnl-2021-320428

		Associated Thoracic Aortic Aneurysm Cohort: SMAD6 as an Important Contributor.	
28	29162281	Targeted next-generation sequencing identified ADAMTS5 as novel genetic substrate in patients with bicuspid aortic valve.	Int J Cardiol. 2018;252:150-155.
29	29332214	Targeted Next-Generation Sequencing in Patients with Non-syndromic Congenital Heart Disease.	Pediatr Cardiol. 2018;39:682-689.
30	29907982	Results of next-generation sequencing gene panel diagnostics including copy-number variation analysis in 810 patients suspected of heritable thoracic aortic disorders.	Hum Mutat. 2018;39:1173-1192.
31	26299364	Heterozygous Loss-of-Function Mutations in DLL4 Cause Adams-Oliver Syndrome.	Am J Hum Genet. 2015; 97: 475–482.
32	29924900	Elucidating the genetic architecture of Adams-Oliver syndrome in a large European cohort.	Hum Mutat. 2018;39:1246-1261.
33	30255099	Bicuspid Aortic Valve: Role of Multiple Gene Variants in Influencing the Clinical Phenotype.	Biomed Res Int. 2018;2018:8386123.
34	30455415	ROBO4 variants predispose individuals to bicuspid aortic valve and thoracic aortic aneurysm.	Nat Genet. 2019;51:42-50.
35	30511478	Loss of function, missense, and intronic variants in NOTCH1 confer different risks for left ventricular outflow tract obstructive heart defects in two European cohorts.	Genet Epidemiol. 2019;43:215-226.
36	30582441	Whole Exome Sequencing Reveals the Major Genetic Contributors to Nonsyndromic Tetralogy of Fallot.	Circ Res. 2019;124:553-563.
37	30848080	A novel SMAD6 variant in a patient with severely calcified bicuspid aortic valve and thoracic aortic aneurysm.	Mol Genet Genomic Med. 2019;7:e620.
38	31111652	Familial aggregation of apple peel interstitial atresia and cardiac left sided obstructive lesions.	Am J Med Genet A. 2019;179:1570-1574.
39	31261205	A novel DLL4 missense mutation in a Chinese patient with Adams-Oliver syndrome.	Chin Med J (Engl). 2019;132:1755-1757.
40	31330235	Sequencing of NOTCH1 gene in an Italian population with bicuspid aortic valve: Preliminary results from the GISSI OUTLIERS VAR study.	Gene. 2019;715:143970.
41	25931334	The diagnostic value of next generation sequencing in familial nonsyndromic congenital heart defects.	Am J Med Genet A. 2015;167A:1822-9.
42	31633846	Contribution of single-gene defects to congenital cardiac left-sided lesions in the prenatal setting.	Ultrasound Obstet Gynecol. 2020;56:225-232.
43	31654484	Expanding the phenotype in Adams-Oliver syndrome correlating with the genotype.	Am J Med Genet A. 2020;182:29-37.
44	31867804	A novel de novo dominant mutation of NOTCH1 gene in an Iranian family with non-syndromic congenital heart disease.	J Clin Lab Anal. 2020;34:e23147.
45	32129674	Novel In-Frame Deletion Mutation in NOTCH1 in a Chinese Sporadic Case of Adams-Oliver Syndrome.	DNA Cell Biol. 2020;39:783-789.
46	32165302	Family-based whole-genome sequencing identifies compound heterozygous protein-coding and noncoding mutations in tetralogy of Fallot.	Gene. 2020;741:144555.
47	32375772	Concurrent pathogenic variants in SLC6A1/NOTCH1/PRIMPOL genes in a Chinese patient with myoclonic-atonic epilepsy, mild aortic valve stenosis and high myopia.	BMC Med Genet. 2020;21:93.
48	32498638	A novel variant in DOCK6 gene associated with Adams-Oliver syndrome type 2.	Ophthalmic Genet. 2020;41:377-380.
49	32544455	Double-hit mutations in bicuspid aortic valve and blunt traumatic acute aortic dissection.	Ann Thorac Surg. 021;111:e5-e6.
50	32720365	Novel loss of function mutation in NOTCH1 in a family with bicuspid aortic valve, ventricular septal defect, thoracic aortic aneurysm, and aortic valve stenosis.	Mol Genet Genomic Med. 2020 ;8:e1437.
51	32748548	Rare deleterious variants of NOTCH1, GATA4, SMAD6, and ROBO4 are enriched in BAV with early onset complications but not in BAV with heritable thoracic aortic disease.	Mol Genet Genomic Med. 2020;8:e1406.
52	32871987	Hypoplastic coronary arteries in a child with a mutation in Notch1: A case report.	Medicine (Baltimore). 2020;99:e21355.

Debiec RM, *et al. Heart* 2022; 108:1114–1120. doi: 10.1136/heartjnl-2021-320428

53	33064175	Recurrent germline mutations as genetic markers for aortic root dilatation in bicuspid aortic valve patients.	Heart Vessels. 2020
54	33110418	Genes and Pathways Implicated in Tetralogy of Fallot Revealed by Ultra-Rare Variant Burden Analysis in 231 Genome Sequences.	Front Genet. 2020;11:957.
55	25085919	Whole Exome Sequencing for Familial Bicuspid Aortic Valve Identifies Putative Variants.	Circulation: Cardiovascular Genetics. 2014;7:677–683.
56	23665959	De novo mutations in histone-modifying genes in congenital heart disease.	Nature. 2013;498:220-3.
57	24702954	Rare variants in NR2F2 cause congenital heart defects in humans.	Am J Hum Genet. 2016;98:592.
58	27479907	Distinct genetic architectures for syndromic and nonsyndromic congenital heart defects identified by exome sequencing. Nat Genet.	Nat Genet. 2016; 48(9):1060-5.
59	22337856	Exome analysis of a family with pleiotropic congenital heart disease.	Circ Cardiovasc Genet. 2012;5: 175–182.
60	25500235	Targeted Next-Generation Sequencing Identifies Pathogenic Variants in Familial Congenital Heart Disease.	J Am Coll Cardiol. 2014;64:2498-506.
61	26854089	Clinically relevant variants identified in thoracic aortic aneurysm patients by research exome sequencing.	Am J Med Genet Part A 170A:1288–1294.
62	26785492	De novo mutations in congenital heart disease with neurodevelopmental and other congenital anomalies.	Science. 2015;35:1262-6.
63	28991257	Contribution of rare inherited and de novo variants in 2,871 congenital heart disease probands.	Nature Genetics.2017;49:1593–1601.
64	29481643	From phenotype to genotype: towards identifying recurrent genetic aberrations in bicuspid aortic valve disease.	Eur J Cardiothorac Surg. 2018;54:198-199.

Debiec RM, *et al. Heart* 2022; 108:1114–1120. doi: 10.1136/heartjnl-2021-320428

Supplementary Table 2. Pathogenic and likely pathogenic NOTCH1 variants identified through literature review.

No	PMID	Nucleotide change	Amino acid change	rs-identifier	gnomAD MAF	Present in Familial/sporadic case	Penetrance	Associated phenotypes	Pathogenicity class
1	16025100	c.3319C>T	p.Arg1107Ter	rs41309764	NA	Familial	Complete	BAV, AS, AI, VSD, ToF, MS	P
2	16025100	c.4512delC	p.Cys1505ValfsTer75	rs41309766	NA	Familial	Complete	BAV, AS, MA, HLHS, DORV	P
3	21457232	c.405delG	p.Lys136AsnfsTer141	NA	NA	Sporadic	NA	HLHS	P
4	23578328	c.4857C>A	p.Tyr1619Ter	NA	NA	Familial	Complete	BAV, AS, CoA	P
5	25132448	c.743-1G>A	NA	NA	NA	familial	Complete	AOS (No cardiac/vascular defects reported)	P
6	25963545	c.1649dupA	p.Tyr550Ter	rs864622059	NA	Familial	Incomplete (6 carriers, 5 affected)	AOS	P
7	25963545	c.6049_6050delTC	p.Ser2017ThrfsTer9	rs864622063	NA	Familial	Complete	AOS	P
8	25963545	c.4663G>T	p.Glu1555Ter	rs746342893	5.07E-05	familial	Complete	AOS	P
9	25963545	c.4739dupT	p.Met1580IlefsTer30	rs864622061	4.92E-06	sporadic	NA	AOS	P
10	26708639	c.1565_1566delGGinsC	p.Gly522AlafsTer109	NA	NA	sporadic	NA	BAV, TAA	P
11	26820064	c.3511-2A>G	NA	NA	NA	familial	Complete (3 carriers/3 affected)	BAV, AS, PVS	P
12	26820064	c.865+2C>A	NA	NA	NA	familial	Incomplete (of 8 mutation carriers 7 was affected)	BAV, AS, PA (pulm atr), TAA, VSD, right sided aorta.	P
13	26820064	c.5950C>T	p.Arg1984Ter	rs1554826746	NA	familial	Complete	BAV, AS, TAA	P
14	26820064	c.2643delC	p.Ala882HisfsTer297	NA	NA	familial	Complete	BAV, AS, TA (truncus	P

Debiec RM, *et al. Heart* 2022; 108:1114–1120. doi: 10.1136/heartjnl-2021-320428

								arterior=sus), PA, VSD, HRV (hypolastic RV), ASD, PDA	
15	26820064	c.5529G>A	p.Trp1843Ter	NA	NA	familial	Complete	BAV, AS, AI, MVS, PA, VSD, MI (mitral valve insufficency)	P
16	26820064	c.4240delT	p.Cys1414AlafsTer31	NA	NA	familial	NA	AS, TAA, ToF, PA	P
17	26820064	c.7455dupC	p.Ser2486LeufsTer21	NA	NA	familial	Complete	BAV, AS, PVS, TA, TAA,	P
18	26820064	c.2425delG	p.Asp809ThrfsTer67	NA	NA	Familial	Incomplete (2 carriers one affected)	BAV, AS, MVS	P
19	26820064	c.1904-2A>G	NA	NA	NA	familial	Incomplete (3 crriers, 2 affected)	BAV, AS, ToF, right-sided aorta.	P
20	26820064	c.3054C>A	p.Cys1018Ter	NA	NA	sporadic	NA	HLHS	P
21	26820064	c.1650C>G	p.Tyr550Ter	NA	NA	familial	Incomplete (14 carriers, 11 affected)	BAV, AS, AI, MVS, ToF, VSD, TAA, TAPVR (total aomalous pulmonary vein return).	P
22	27760138	c.3765C>A	p.Cys1255Ter	rs1057515423	NA	familial	Incomplete (8 carriers/ 7 affected)	BAV, AS, ToF	P
23	27760138	c.2439C>G	p.Tyr813Ter	rs1057515422	NA	familial	Complete	BAV, AS	P
24	28473349	c.5767delC	p.Gln1923ArgfsTer58	NA	NA	familial	Complete	HLHS, VSD, ToF, AS, MA, DORV	P
25	28608148	c.4662C>A	p.Cys1554Ter	NA	4.08E-06	familial	Incomplete (2 carriers/1	HLHS	P

							affected)		
26	29924900	c.415C>T	p.Gln139Ter	rs1554730670	NA	Familial	Incomplete (3 carriers/2 affected)	AOS	P
27	29924900	c.794_797delACTGinsCC	p.Asn265ThrfsTer65	rs1554730184	NA	Familial	Complete (2 carriers/2 affected)	AOS	P
28	29924900	c.2380G>T	p.Glu794Ter	rs1554729113	NA	Familial	Incomplete (2 carriers 1 affected)	AOS	P
29	29924900	c.4222G>T	p.Glu1408Ter	rs587778569	2.05E-05	sporadic	NA	AOS	P
30	30511478	c.1651_1653delACCinsTAA	p.Thr551Ter	NA	NA	Familial	Incomplete (2 carriers 1 affected)	HLHS, CoA	P
31	30511478	c.2741-1G>A	NA	NA	NA	Familial	Incomplete (2carriers/1 affected)	HLHS	P
32	30582441	c.5197C>T	p.Gln1733Ter	NA	NA	sporadic	NA	ToF	P
33	30582441	c.4913G>A	p.Trp1638Ter	NA	NA	sporadic	NA	ToF	P
34	30582441	c.1342C>T	p.Arg448Ter	rs869025494	NA	sporadic	NA	ToF	P
35	30582441	c.5385-2delA	NA	NA	NA	sporadic	NA	ToF	P
36	30582441	c.3966delC	p.Cys1322TrpfsTer123	NA	NA	sporadic	NA	ToF	P
37	30582441	c.440delA	p.Asn147ThrfsTer130	NA	NA	sporadic	NA	ToF	P
38	25931334	c.5281delC	p.Arg1761GlyfsTer37	rs515726231	NA	familial	Incomplete (6 carriers/3 affected)	HLHS, CoA, AS, TOF, DORV, MS	P
39	25931334	c.2014+1G>A	NA	rs515726232	NA	familial	Incomplete (3 carriers/2 afftetced)	HLHS, PDA, DORV, TGA, PS, ASD	P
40	31633846	c.3643+1G>A	NA	NA	NA	sporadic	NA	CoA	P
41	31633846	c.4015-2A>G	NA	NA	NA	sporadic	NA	HLHS	P

Debiec RM, *et al. Heart* 2022; 108:1114–1120. doi: 10.1136/heartjnl-2021-320428

42	31633846	c.4837C>T	p.Gln1613Ter	NA	NA	sporadic	NA	congenital AS	P
43	31633846	c.2452dupC	p.Leu818ProfsTer10	NA	NA	sporadic	NA	HLHS	P
44	31654484	c.2346delC	p.Phe783SerfsTer19	NA	NA	Familial	Incomplete (2 carriers/1 affected)	AOS (VSD, ASD, AOS)	P
45	31654484	c.3319C>T	p.Arg1107Ter	rs41309764	NA	sporadic	NA	AOS (COA, ASD)	P
46	32375772	c.1100-2A>G	NA	NA	NA	Sporadic	NA	Myoclonic atonic epilepsy, learning difficulty, AS, myopia	P
47	32720365	c.873C>G	p.Tyr291Ter	NA	NA	Familial	Complete	BAV, AS, TAA, VSD	P
48	32748548	c.2524_2525delGGinsTA	p.Gly842Ter	NA	NA	sporadic	NA	BAV, TAA	P
49	27479907	c.1530delT	p.Asn510LysfsTer121	NA	NA	Familial	NA	CHD	P
50	27479907	c.4563C>A	p.Cys1521Ter	NA	NA	Familial	NA	CHD	P
51	27479907	c.5197C>T	p.Gln1733Ter	rs1208976166	NA	Familial	NA	CHD	P
52	27479907	c.5385-2delA	NA	Na	NA	sporadic	NA	CHD	P
53	26785492	c.2207+1G>T	NA	NA	NA	sporadic	NA	Shone's syndrome (BAV, CoA, abnormal MV, hypolpastic aorta)	P
54	28991257	c.5318dupT	p.Lys1774GlnfsTer4	NA	NA	sporadic	NA	ToF	P
55	28991257	c.645C>A	p.Cys215Ter	NA	NA	sporadic	NA	Subvalvular AS, hypolastic aorta, CoA, abnormal MV	P
56	28991257	c.4563C>A	p.Cys1521Ter	NA	NA	sporadic	NA	HLHS, Hypoplastic aorta, MS	P
57	28991257	c.1529_1530insG	p.Asn510LysfsTer2	NA	NA	sporadic	NA	Aortic atresia, HLHS, Mitral	P

Debiec RM, *et al. Heart* 2022; 108:1114–1120. doi: 10.1136/heartjnl-2021-320428

								atresia , LSVC	
58	28991257	c.273_277delGGGCT	p.Gly92LeufsTer49	NA	NA	sporadic	NA	ToF, PA, MAPCAS	P
59	28991257	c.1342C>T	p.Arg448Ter	rs869025494	NA	sporadic	NA	CoA, Hypoplastic LV, MS	P
60	28991257	c.1800_1801dupCG	p.Glu601AlafsTer31	NA	NA	sporadic	NA	ToF, ASD,	P
61	28991257	c.3055C>T	p.Gln1019Ter	NA	NA	sporadic	NA	DORV, Hypoplastic LV, mitral atresia	P
62	26820064	c.3787C>T	p.Arg1263Cys	rs1415111675	4.1E-06	familial	Incomplete (6 carriers/2 affetced)	BAV, AS, MVS, CoA, PDA, LV non-compaction	P
63	29924900	c.1935_1936delTG	p.Ala646GlnfsTer21	rs1554729443	NA	Familial	Complete (3 carriers/3 affected)	AOS	P
64	30511478	c.1077C>A	p.Cys359Ter	NA	NA	sporadic	NA	HLHS	P
65	30582441	c.344delG	p.Gly115AlafsTer8	NA	NA	sporadic	NA	ToF	P
66	25132448	c.1285T>C	p.Cys429Arg	rs587777736	NA	sporadic	NA	AOS (No cardiac/vascular defects reported)	LP
67	25132448	c.4487G>A	p.Cys1496Tyr	rs587781259	NA	sporadic	NA	AOS (Mild narrowing of arotich arch, multiperforate PFO)	LP
68	25963545	c.1345T>C	p.Cys449Arg	rs864622057	NA	sporadic	NA	AOS	LP
69	25963545	c.1367G>A	p.Cys456Tyr	rs864622058	NA	sporadic	NA	AOS	LP
70	27760138	c.578G>A	p.Gly193Asp	rs774966208	NA	familial	Complete	TOF, VSD	LP
71	29924900	c.4549G>A	p.Asp1517Asn	rs1554727954	NA	sporadic	NA	AOS	LP
72	32129674	c.1292_1294delACA	p.Asn431del	NA	NA	Sporadic	NA	AOS	LP

Debiec RM, *et al. Heart* 2022; 108:1114–1120. doi: 10.1136/heartjnl-2021-320428

73	26785492	c.4549G>A	p.Asp1517Asn	rs1554727954	NA	sporadic	NA	HLHS, hypoplastic aorta, ASD	LP
74	30582441	c.4646G>A	p.Cys1549Tyr	NA	NA	sporadic	NA	ToF	LP
75	30582441	c.1820G>A	Cys607Tyr	NA	NA	sporadic	NA	ToF	LP
76	30582441	c.599G>T	p.Gly200Val	NA	NA	sporadic	NA	ToF	LP
77	30582441	c.598G>C	p.Gly200Arg	NA	NA	sporadic	NA	ToF	LP
78	30582441	c.578G>C	p.Gly193Ala	rs774966208	NA	sporadic	NA	ToF	LP
79	30582441	c.428C>T	p.Pro143Leu	rs1228192276	NA	sporadic	NA	ToF	LP
80	31654484	1364A>T	p.Glu455Val	NA	NA	Familial	NA	AOS (Pulmonary HTN)	LP
81	27479907	c.4646G>A	p.Cys1549Tyr		NA	sporadic	NA	CHD	LP
82	25500235	c.598G>C	p.Gly200Arg	NA	NA	Familial	NA	ToF, PTA, VSD, PA, ALCAPA	LP
83	25963545	c.4120T>C	p.Cys1374Arg	rs864622060	NA	familial	Incomplete (3 carriers two affected)	AOS	US
84	29924900	c.1393G>A	p.Ala465Thr	rs1057523819	NA	sporadic	NA	AOS	US
85	29924900	c.2704C>T	p.Arg902Cys	rs1448345366	4.17E-06	sporadic	NA	AOS	US
86	29924900	c.3281G>A	p.Cys1094Tyr	rs1554728424	NA	sporadic	NA	AOS	US
87	30582441	c.5017G>C	p.Gly1673Arg	NA	NA	sporadic	NA	ToF	US
88	30582441	c.1441G>A	p.Gly481Ser	rs1448146225	4.06E-06	sporadic	NA	ToF	US
89	28991257	c.1253T>C	p.Leu418Pro	NA	NA	sporadic	NA	ToF, ASD, PA	US
90	21457232	c.3239A>G	p.Gln1080Arg	NA	0.000032	Familial	Complete	HLHS	US
91	21457232	c.4427G>A	p.Gly1476Asp	rs1460297840	0.000004	sporadic	NA	HLHS	US
92	21457232	c.4477T>C	p.Ser1493Pro	rs749283347	0.000012	Sporadic	NA	HLHS	US

93	23102684	c.3269C>G	p.Thr1090Ser	rs761508282	0.000011	Sporadic	NA	BAV, TAA	US
94	25963545	c.1220C>G	p.Pro407Arg	rs754529382	0.000014	sporadic	NA	AOS	US
95	26164125	c.5891C>T	p.Pro1964Leu	rs1387329667	NA	Familial	NA	CHD (HLHS, BAV, Bicuspid pulmonary valve)	US
96	26820064	c.136T>G	p.Cys46Gly	NA	NA	sporadic	NA	CoA	US
97	26820064	c.1030T>A	p.Cys344Ser	NA	NA	sporadic	NA	HLHS	US
98	26820064	c.3271G>A	p.Gly1091Ser	rs768095251	0.000056	familial	Incomplete (3 cariiers/2 affected)	BAV, HLHS,	US
99	26820064	c.4382A>G	p.Lys1461Arg	NA	NA	Familial	NA	BAV	US
100	26820064	c.5006T>G	p.Met1669Arg	NA	NA	familial	NA	BAV	US
101	28246602	c.3913G>A	p.Glu1305Lys	rs775602958	NA	sporadic	NA	BAV, AS	US
102	28246602	c.3799G>A	p.Asp1267Asn	NA	NA	sporadic	NA	BAV, AS	US
103	28387797	c.1334C>T	p.Thr445Met	rs752919688	0.000012	sporadic	NA	VSD, AOS	US
104	28649221	c.6365C>T	p.Pro2122Leu	rs767587816	8.3E-06	sporadic	NA	Middle size arteries dissections, TAA	US
105	28659821	c.982A>T	p.Thr328Ser	NA	NA	sporadic	NA	BAV, TAA	US
106	28659821	c.1951G>A	p.Asp651Asn	rs1182867386	8.1E-06	sporadic	NA	BAV, TAA	US
107	28659821	c.4013C>T	p.Ala1338Val	rs1397249771	0.000014	sporadic	NA	BAV, TAA	US
108	28659821	c.5047C>T	p.Arg1683Trp	rs755746883	0.000004	sporadic	NA	BAV, TAA	US
109	28659821	c.6413C>T	p.Pro2138Leu	rs756571156	8.4E-06	sporadic	NA	BAV, TAA	US
110	29332214	c.6949G>T	p.Gly2317Cys	NA	NA	sporadic	NA	PS	US
111	29907982	c.2123A>G	p.Tyr708Cys	NA	NA	sporadic	NA	TAA	US
112	29924900	c.4240T>A	p.Cys1414Ser	NA	NA	Familial	Incomplete 6carriers/3 affected)	AOS	US

Debiec RM, *et al. Heart* 2022; 108:1114–1120. doi: 10.1136/heartjnl-2021-320428

113	29924900	c.1669+5G>A	NA	rs771590616	NA	Familial	incomplete (3 carriers / 2 affected)		US
114	29924900	c.5272C>G	p.Arg1758Gly	rs777859108	4.2E-06	Familial	Complete	AOS	US
115	29924900	c.6100T>A	p.Trp2034Arg	NA	NA	sporadic	NA	AOS	US
116	29924900	c.6128C>T	p.Ala2043Val	rs1554826688	NA	sporadic	NA	AOS	US
117	29924900	c.1582G>A	p.Asp528Asn	rs757988142	8.1E-06	sporadic	NA	AOS	US
118	30582441	c.6011G>T	p.Arg2004Leu	NA	NA	sporadic	NA	ToF	US
119	30582441	c.5624A>G	p.Asn1875Ser	NA	NA	sporadic	NA	ToF	US
120	30582441	c.5497G>C	p.Asp1833His	NA	NA	sporadic	NA	ToF	US
121	30582441	c.4483C>A	p.Gln1495Lys	rs1036363986	NA	sporadic	NA	ToF	US
122	30582441	c.4025G>A	p.Gly1342Asp	rs775187426	9.6E-06	sporadic	NA	ToF	US
123	30582441	c.3974C>T	p.Ala1325Val	NA	8.4E-06	sporadic	NA	ToF	US
124	30582441	c.3880G>A	p.Glu1294Lys	rs1247035429	4.5E-06	sporadic	NA	ToF	US
125	30582441	c.1934G>A	p.Cys645Tyr	NA	0.000004	sporadic	NA	ToF	US
126	30582441	c.1412T>A	p.Ile471Asn	NA	NA	sporadic	NA	ToF	US
127	30582441	c.1057C>T	p.Arg353Cys	rs1300110216	0.000032	sporadic	NA	ToF	US
128	30582441	c.875G>A	p.Cys292Tyr	NA	NA	sporadic	NA	ToF	US
129	30582441	c.545G>A	p.Cys182Tyr	NA	NA	sporadic	NA	ToF	US
130	30582441	c.490T>G	p.Cys164Gly	NA	NA	sporadic	NA	ToF	US
131	30582441	c.214G>A	p.Gly72Arg	rs892114222	8.8E-06	sporadic	NA	ToF	US
132	30582441	c.4428_4430delCGG	p.Gly1477del		NA	sporadic	NA	ToF	US
133	30582441	c.436_450dupTCCAACCCCTGCGCC	p.Ser146_Ala150dup		NA	sporadic	NA	ToF	US
134	31330235	c.1619G>A	p.Gly540Asp	rs767618789	8.1E-06	sporadic	NA	BAV	US
135	31330235	c.2551G>C	p.Glu851Gln	NA	NA	sporadic	NA	BAV	US

Debiec RM, *et al. Heart* 2022; 108:1114–1120. doi: 10.1136/heartjnl-2021-320428

136	25931334	c.5061G>C	p.Gln1687His	NA	NA	familial	NA	HLHS, AS, CoA	US
137	31633846	c.3973G>A	p.Ala1325Thr	rs767910007	0.000041	sporadic	NA	HLHS	US
138	31654484	c.1246T>A	p.Cys416Ser	NA	NA	Familial	NA	AOS (PS)	US
139	31654484	c.1732G>C	p.Gly578Arg	NA	NA	Familial	NA	AOS (ASD)	US
140	32165302	c.2054A>T	p.Asn685Ile	NA	NA	Sporadic	NA	ToF	US
141	32748548	c.1757C>T	p.Thr586Ile	NA	NA	sporadic	NA	BAV, TAA	US
142	32748548	c.3068A>G	p.Asn1023Ser	rs937138982	6.1E-06	sporadic	NA	BAV, TAA	US
143	32748548	c.3397T>G	p.Cys1133Gly	NA	NA	sporadic	NA	BAV, TAA	US
144	32748548	c.4010C>G	p.Pro1337Arg	rs1043832212	NA	sporadic	NA	BAV, TAA	US
145	32748548	c.4718C>T	p.Thr1573Met	rs573864607	0.000026	sporadic	NA	BAV, TAA	US
146	32748548	c.6365C>T	p.Pro2122Leu	rs767587816	8.3E-06	sporadic	NA	BAV, TAA	US
147	32748548	c.2806G>A	p.Gly936Ser	rs773847667	0.000016	sporadic	NA	BAV, TAA	US
148	32871987	c.1023C>A	p.Ser341Arg	NA	NA	sporadic	NA	Hypoplastic coronary arteries	US
149	33064175	c.3679C>T	p.Pro1227Ser	rs777652834	0.000075	sporadic	NA	BAV, TAA	US
150	33110418	c.2444G>A	p.Cys815Tyr	NA	NA	sporadic	NA	ToF, PA, Right aortic arch	US
151	33110418	c.1816G>A	p.Glu606Lys	rs1381792229	NA	Familial	NA	ToF	US
152	33110418	c.2045G>A	p.Cys682Tyr	NA	NA	sporadic	NA	ToF	US
153	33110418	c.1243G>A	p.Glu415Lys	NA	NA	Familial	NA	ToF	US
154	33110418	c.4606T>C	p.Cys1536Arg	NA	NA	Familial	NA	ToF, HLHS, BAV, CoA	US
155	33110418	c.1869C>A	p.Asn623Lys	NA	NA	sporadic	NA	ToF, Right aortic arch, absent pulmonary valve, learning difficulties	US
156	33110418	c.2128G>A	p.Asp710Asn	rs950236535	NA	sporadic	NA	ToF	US

Debiec RM, *et al. Heart* 2022; 108:1114–1120. doi: 10.1136/heartjnl-2021-320428

157	33110418	c.847T>G	p.Cys283Gly	NA	NA	Familial	NA	TOF	US
158	27479907	c.273_278delGGGCTT	p.Gly92_Phe93del		NA	Familial	NA	CHD	US
159	25500235	c.3271G>A	p.Gly1091Ser	rs768095251	0.000056	Familial	NA	CHD	US
160	25500235	c.4078G>A	p.Gly1360Ser	rs769493139	0.000012	Familial	NA	CHD	US
161	28991257	c.1430T>A	p.Ile477Asn	NA	NA	sporadic	NA	ToF	US
162	28991257	c.961T>G	p.Cys321Gly	NA	NA	sporadic	NA	BAV, ASD, VSD	US
163	17662764	c.4049G>T	p.Arg1350Leu	rs150343794	9.1E-06	Sporadic	NA	BAV, TAA	LB
164	18593716	c.1981G>A	p.Gly661Ser	rs201077220	0.00036	Sporadic	NA	BAV, CoA, AS, HLHS	LB
165	18593716	c.4049G>T	p.Arg1350Leu	rs150343794	9.1E-06	Sporadic	NA	AS	LB
166	23578328	c.851C>T	p.Pro284Leu	rs376104770	0.000093	Familial	complete	BAV, TAA	LB
167	25260786	c.6481C>T	p.Pro2161Ser	rs201518848	0.000047	sporadic	NA	BAV	LB
168	25907466	c.157G>A	p.Val53Met	rs757497167	0.00004	Familial	Complete	BAV/TAA	LB
169	25907466	c.3706C>T	p.Pro1236Ser	rs749988739	0.000021	sporadic	NA	BAV/TAA	LB
170	26708639	c.4472C>T	p.Thr1491Met	rs369915496	0.00002	sporadic	NA	BAV, TAA	LB
171	26708639	c.454G>A	p.Gly152Ser	rs750242131	0.00005	sporadic	NA	BAV, TAA	LB
172	26820064	c.839A>G	p.Asn280Ser	rs367825691	0.00028	sporadic	NA	CoA	LB
173	26820064	c.1801G>A	p.Glu601Lys	rs749381544	0.000051	sporadic	NA	AS	LB
174	26820064	c.2003C>T	p.Pro668Leu	rs576030298	0.000039	sporadic	NA	BAV, TAA	LB
175	26820064	c.2636G>A	p.Arg879Gln	rs368011392	0.000091	sporadic	NA	HLHS	LB
176	26820064	c.3328G>A	p.Val1110Ile	rs778969906	4.1E-06	sporadic	NA	CaA	LB
177	26820064	c.3859C>T	p.Arg1287Cys	rs751275854	0.000017	sporadic	NA	HLHS	LB
178	26820064	c.4031C>T	p.Thr1344Met	rs201215245	0.00002	familial	Incomplete (3 carriers/2 affected)	BAV, HLHS,	LB
179	26820064	c.4049G>T	p.Arg1350Leu	rs150343794	9.1E-06	Familial	Incomplete (4 carriers/3	HLHS, CoA, VSD,	LB

							affected)		
180	26820064	c.4969A>C	p.Ser1657Arg	NA	NA	sporadic	NA	BAV	LB
181	26820064	c.6938G>A	p.Arg2313Gln	rs371069660	0.000086	sporadic	NA	HLHS	LB
182	26820064	c.7397C>T	p.Thr2466Met	rs369167555	0.000042	sporadic	NA	HLHS	LB
183	26820064	c.7432G>A	p.Ala2478Thr	rs779039862	0.00002	sporadic	NA	HLHS	LB
184	27760138	c.3860G>A	p.Arg1287His	rs763679772	0.000064	familial	NA	TOF, VSD	LB
185	27989580	c.3835C>T	p.Arg1279Cys	rs182330532	0.00038	Familial	Incomplete (5 carriers/4 affected)	ASD, PFO	LB
186	28387797	c.4492A>G	p.Lys1498Glu	rs745681787	0.000043	sporadic	NA	BAV, TAA	LB
187	28387797	c.5414T>C	p.Leu1805Pro	rs201779159	0.000054	sporadic	NA	BAV, TAA	LB
188	28659821	c.4021G>A	p.Glu1341Lys	rs372767143	4.9E-06	sporadic	NA	BAV, TAA	LB
189	28659821	c.5248G>A	p.Val1750Met	rs368396893	4.2E-06	sporadic	NA	BAV, TAA	LB
190	28659821	c.5414T>C	p.Leu1805Pro	rs201779159	0.000054	sporadic	NA	BAV, TAA	LB
191	29162281	c.4297G>A	p.Gly1433Arg	rs751904604	4.1E-06	sporadic	NA	BAV, AS	LB
192	29924900	c.5218G>T	p.Ala1740Ser	rs864622062	NA	sporadic	NA	AOS	LB
193	29924900	c.5452C>G	p.Leu1818Val	rs1064796983	NA	sporadic	NA	AOS	LB
194	30255099	c.5215G>A	p.Val1739Met	rs377294245	0.000019	Familial	NA	BAV, TAA	LB
195	30511478	c.7178A>G	p.Gln2393Arg	NA	NA	sporadic	NA	HLHS, BAV, CoA, HAA, ASD, VSD	LB
196	30511478	c.3853G>A	p.Val1285Met	rs756972680	0.000013	sporadic	NA	HLHS	LB
197	25931334	c.5332G>C	p.Ala1778Pro	rs986394043	NA	familial	NA	ASD, AVSD, IAA	LB
198	25931334	c.4918G>A	p.Ala1640Thr	rs976118697	0.000016	familial	NA	ASD, AVSD, IAA	LB
199	25931334	c.6685G>A	p.Val2229Met	rs202096917	0.00017	familial	NA	TOF	LB
200	25931334	c.580A>C	p.Thr194Pro	rs770333242	NA	familial	NA	HLHS, CoA, AS, TOF, DORV, MS	LB

Debiec RM, *et al. Heart* 2022; 108:1114–1120. doi: 10.1136/heartjnl-2021-320428

201	32748548	c.383G>A	p.Arg128His	rs754086897	8.4E-06	sporadic	NA	BAV, TAA	LB
202	32748548	c.1870G>A	p.Ala624Thr	rs372771179	0.000033	sporadic	NA	BAV, TAA	LB
203	32748548	c.2675G>A	p.Arg892His	rs199506721	8.4E-06	sporadic	NA	BAV, TAA	LB
204	32748548	c.3835C>T	p.Arg1279Cys	rs182330532	0.00038	sporadic	NA	BAV, TAA	LB
205	32748548	c.4315G>A	p.Asp1439Asn	rs200232299	0.000026	sporadic	NA	BAV, TAA	LB
206	32748548	c.5438G>T	p.Trp1813Leu	rs755659037	0.000043	sporadic	NA	BAV, TAA	LB
207	32748548	c.7115G>A	p.Arg2372Gln	rs373119531	0.0001	sporadic	NA	BAV, TAA	LB
208	25500235	c.5438G>C	p.Trp1813Ser	rs755659037	0.000043	Familial	NA	CHD	LB
209	28991257	c.851C>T	p.Pro284Leu	rs376104770	0.000093	sporadic	NA	CoA, abnomral MV, ASD	LB
210	18593716	c.2047G>A	p.Ala683Thr	rs756434709	0.00002	Sporadic	NA	AS, HLHS	LB
211	16729972	c.1787C>T	p.Thr596Met	rs61755997	0.00017	Sporadic	NA	BAV, TAA	B
212	17662764	c.4168C>A	p.Pro1390Thr	rs191645600	0.00056	Sporadic	NA	BAV, TAA	B
213	18593716	c.2080G>A	p.Glu694Lys	rs79782048	0.00064	Sporadic	NA	AS	B
214	18593716	c.7606G>A	p.Val2536Ile	rs111627256	0.00039	Sporadic	NA	BAV, CoA	B
215	25132448	c.5965G>A	p.Asp1989Asn	rs587777734	NA	Familial	NA	AOS (No cardiac defects in the proband)	B
216	26164125	c.3767C>T	p.Pro1256Leu	rs80340744	4.1E-06	Familial	NA	CHD (HLHS, BAV, Bicuspid pulmonary valve)	B
217	26820064	c.701G>A	p.Arg234His	rs150737112	0.00045	sporadic	NA	AVS	B
218	26820064	c.2080G>A	p.Glu694Lys	rs79782048	0.00064	sporadic	NA	BAV	B
219	26820064	c.7606G>A	p.Val2536Ile	rs111627256	0.00039	sporadic	NA	HLHS	B
220	28387797	c.4168C>A	p.Pro1390Thr	rs191645600	0.00056	sporadic	NA	BAV, TAA	B
221	29924900	c.6788G>A	p.Arg2263Gln	rs200521815	0.00036	sporadic	NA	AOS	B
222	31330235	c.4313G>A	p.Arg1438His	rs61751541	0.00011	sporadic	NA	BAV, TAA	B

Debiec RM, *et al. Heart* 2022; 108:1114–1120. doi: 10.1136/heartjnl-2021-320428

223	31867804	c.6797T>C	p.Phe2266Ser	NA	NA	Familial	complete	VSD, PDA, AVSD	B
224	32748548	c.701G>A	p.Arg234His	rs150737112	0.00045	sporadic	NA	BAV, TAA	B
225	32748548	c.1511G>A	p.Arg504His	rs201768800	0.00033	sporadic	NA	BAV, TAA	B
226	32748548	c.4898G>A	p.Arg1633His	rs375018022	0.00016	sporadic	NA	BAV, TAA	B
227	25500235	c.1787C>T	p.Thr596Met	rs61755997	0.00017	Familial	NA	CHD	B

MAF – Minor allele frequency, P – pathogenic, LP – Likely pathogenic, AI –aortic valve insufficiency, AOS – Adams Oliver syndrome AS – aortic valve stenosis, ASD- atrial septal defect, BAV – bicuspid aortic valve, CoA – coarctation of aorta, CHD - unspecified congenital heart disease, DORV – double outlet right ventricle, HLHS – hypoplastic left heart syndrome, HRV – hypoplastic right ventricle, MA, mitral atresia, MS – mitral valve stenosis, PA – pulmonary atresia, PDA – patent ductus arteriosus, PS – pulmonary valve stenosis, TA – tricuspid atresia, TAA – thoracic aortic aneurysm, TAPVR – total anomalous pulmonary venous return, TGA – transposition of great arteries, ToF – tetralogy of Fallot, TrA – truncus arteriosus, VSD - ventricular septal defect

Debiec RM, *et al. Heart* 2022; 108:1114–1120. doi: 10.1136/heartjnl-2021-320428

Supplementary Figure 1. Schematic location of identified pathogenic and likely pathogenic mutations in *NOTCH1*

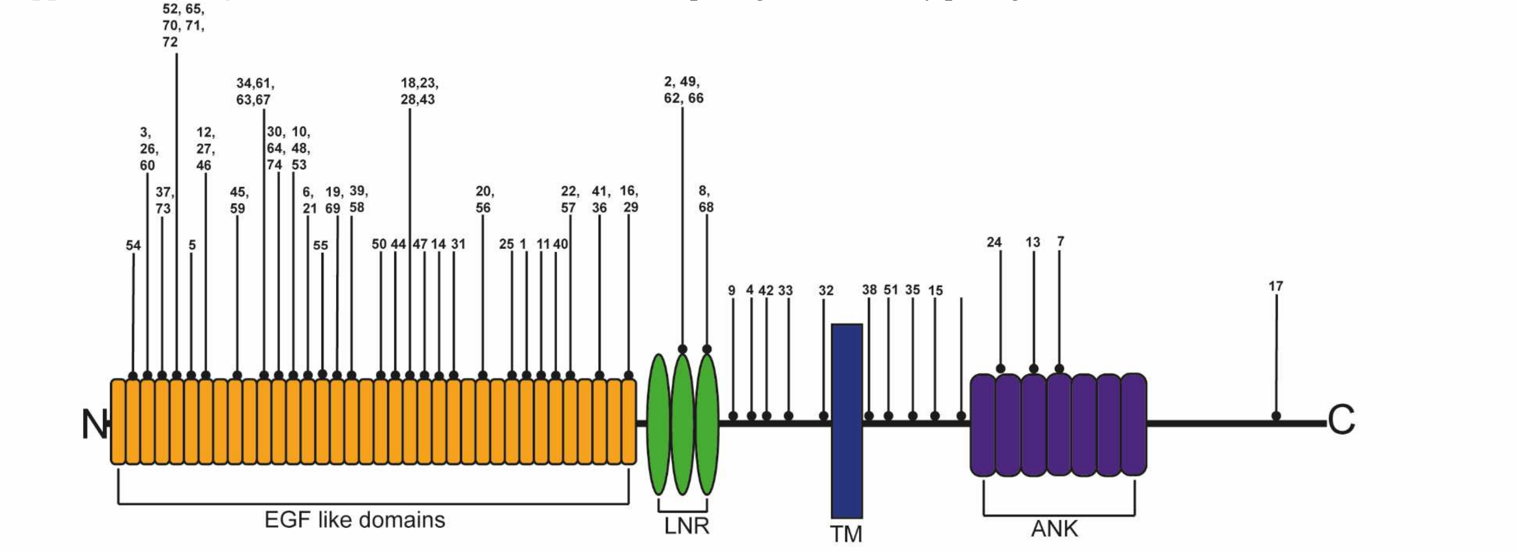

1 - c.3319C>T (p.Arg1107Ter)
2 - c.4512del (p.Cys1505fs)
3 - c.405delG 9 (p.Lys136AsnfsTer141)
4 - c.4857C>A (p.Y1619X)
5 - c.743-1G>A
6 - c.1649dupA (p.Tyr550Ter)
7 - c.6049_6050delTC (p.Ser2017ThrfsTer9)
8 - c.4663G>T (p.Glu1555Ter)
9 - c.4739dupT (p.Met1580IlefsTer30)
10 - p.Gly522AlafsTer109
11 - c.3511-2A>G
12 - c.865+2C>A
13 - c.5950C>T (p.Arg1984Ter)
14 - c.2643delC (p.Ala882HisfsTer297)
15 - c.5529G>A (p.Trp1843Ter)
16 - c.4240delT (p.Cys1414AlafsTer31)
17 - c.7455dupC (p.Ser2486LeufsTer21)
18 - c.2425delG (p.Asp809ThrfsTer67)
19 - c.1904-2A>G
20 - c.3054C>A (p.Cys1018Ter)
21 - c.1650C>G (p.Tyr550Ter)
22 - c.3765C>A (p.Cys1255Ter)
23 - c.2439C>G (p.Tyr813Ter)
24 - c.5767delC (p.Gln1923ArgfsTer58)
25 - c.4662C>A (p.Cys1554Ter)
26 - c.415C>T (p.Gln139Ter)
27 - c.794_797delACTGinsCC (p.Asn265ThrfsTer65)
28 - c.2380G>T (p.Glu794Ter)
29 - c.4222G>T (p.Glu1408Ter)
30 - c.1651_1653delACCinsTAA (p.Thr551Ter)
31 - c.2741-1G>A
32 - c.5197C>T (p.Gln1733Ter)
33 - c.4913G>A (p.Trp1638Ter)
34 - c.1342C>T (p.Arg448Ter)
35 - c.5385-2delA
36 - c.3966delC (p.Cys1322TrpfsTer123)
37 - c.440delA (p.Asn147ThrfsTer130)
38 - c.5281delC (p.Arg1761GlyfsTer37)
39 - c.2014+1G>A
40 - c.3643+1G>A
41 - c.4015-2A>G
42 - c.4837C>T (p.Gln1613Ter)
43 - c.2452dupC (p.Leu818ProfsTer10)
44 - c.2346delC (p.Phe783SerfsTer19)
45 - c.1100-2A>G
46 - c.873C>G (p.Tyr291Ter)
47 - c.2524_2525delGGinsTA (p.Gly842Ter)
48 - c.1530delT (p.Asn510LysfsTer121)
49 - c.4563C>A (p.Cys1521Ter)
50 - c.2207+1G>T
51 - c.5318dupT (p.Lys1774GlnfsTer4)
52 - c.645C>A (p.Cys215Ter)
53 - c.1529_1530insG (p.Asn510LysfsTer2)
54 - c.273_277delGGGCT (p.Gly92LeufsTer49)
55 - c.1800_1801dupCG (p.Glu601AlafsTer31)
56 - c.3055C>T (p.Gln1019Ter)
57 - c.3787C>T (p.Arg1263Cys)
58 - c.1935_1936delTG (p.Ala646GlnfsTer21)
59 - c.1077C>A (p.Cys359Ter)
60 - c.344delG (p.Gly115AlafsTer8)
61 - c.1285T>C (p.Cys429Arg)
62 - c.4487G>A (p.Cys1496Tyr)
63 - c.1345T>C (p.Cys449Arg)
64 - c.1367G>A (p.Cys456Tyr)
65 - c.578G>A (p.Gly193Asp)
66 - c.4549G>A (p.Asp1517Asn)
67 - c.1292_1294delACA (p.Asn431del)
68 - c.4646G>A (p.Cys1549Tyr)
69 - c.1820G>A (p.Cys607Tyr)
70 - c.599G>T (p.Gly200Val)
71 - c.598G>C (p.Gly200Arg)
72 - c.578G>C (p.Gly193Ala)
73 - c.428C>T (p.Pro143Leu)
74 - 1364A>T (p.Glu455Val)

Debiec RM, *et al. Heart* 2022; 108:1114–1120. doi: 10.1136/heartjnl-2021-320428

EGF- epidermal growth factor-like domain, LNR – Lin12/Notch domains, TM – transmembrane segment, ANK – ankyrin repeats

Debiec RM, *et al. Heart* 2022; 108:1114–1120. doi: 10.1136/heartjnl-2021-320428
